# Supplementary material for: Line excitation array detection fluorescence microscopy at 0.8 million frames per second
Source: Nat Commun. 2018 Oct 29;9:4499. doi: 10.1038/s41467-018-06775-0 (PMC6206139; doi:10.1038/s41467-018-06775-0)
Supplement: Supplementary file 1 — Supplementary Information [file 41467_2018_6775_MOESM1_ESM.pdf]

Supplementary Information

Line excitation array detection fluorescence microscopy at 0.8 million frames per second

Chris Martin[1], Tianqi Li[2], Evan Hegarty[2], Peisen Zhao[3], Sudip Mondal[2], and Adela Ben-Yakar[1-3]

1. Department of Biomedical Engineering, The University of Texas at Austin, 107 W Dean Keeton St., Austin, Texas, USA, 78712.

2. Department of Mechanical Engineering, The University of Texas at Austin, 204 E Dean Keeton St., Austin, Texas, USA, 78712.

3. Department of Electrical and Computer Engineering, The University of Texas at Austin, 2501 Speedway, Austin, Texas, USA, 78712.

Correspondence should be addressed to A.B. (ben-yakar@mail.utexas.edu).

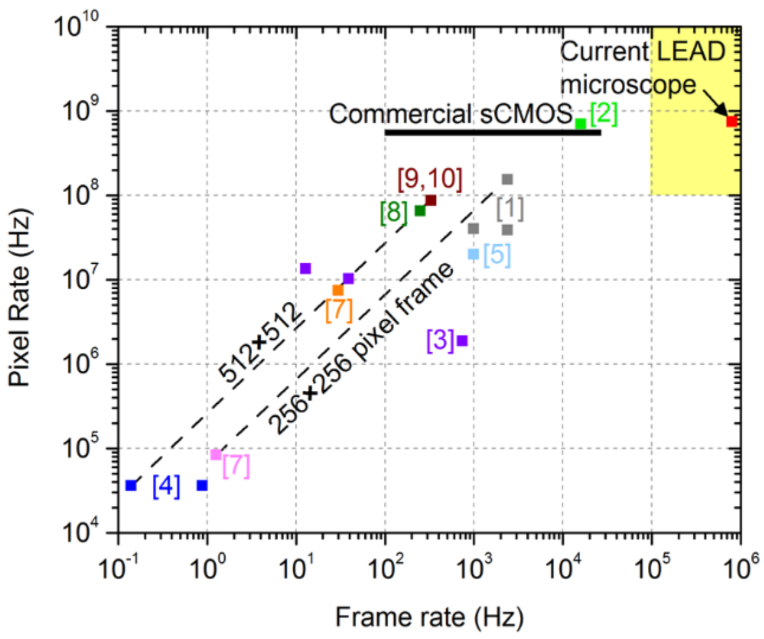

Supplementary Figure 1 | Summary of imaging speeds for fluorescence imaging technologies (see **Supplementary Table 1** for details). The state-of-the-art fluorescence imaging technologies are limited to 2.4 kHz frame rate using a sCMOS camera[1], and 16 kHz frame rate using a PMT with multiplexing[2], while our current LEAD microscope provides 0.8 MHz frame rate and the highest pixel rate. Future LEAD systems can potentially image larger field of views with larger frame sizes than the current system, and reach 10 GHz pixel rates with a 256 element PMT. The highlighted region is the LEAD microscopy regime.

The previous fastest imaging system uses multiplexing, a single PMT, and an 8 kHz resonant mirror to reach 16 kHz frame rates and 702 MHz pixel rates with 190×231 pixel frames[2]. However, since all the pixels along one dimension are captured simultaneously using a single PMT, the dynamic range per pixel is reduced, and all the pixels share increased shot noise, creating a theoretical limit to their maximum achievable frame-rates. On the other hand, LEAD microscopy maintains high dynamic range and low noise per pixel. The frame sizes in Mikami et al.[2] are larger than the current LEAD system, making it more suitable for high resolution or large FOV imaging, albeit at lower frame-rates. Future LEAD systems can have frames with more pixels using higher bandwidth or larger aperture AODs and using PMTs with more elements.

Compared to the fastest camera-based imaging method (SCAPE) of Bouchard et al.[1], LEAD has smaller frames but much higher frame rate. Current sCMOS cameras are limited in their maximum frame rate, even when using a small region of interest, as seen by the commercial sCMOS range in the figure. Faster frame rates would require new cameras with faster readout rates. On the other hand, LEAD can continually improve on the frame size while maintaining high frame rates.

Ref #	Author	Description of imaging method	Pixels per frame	Time per pixel	Switching time	Frame rate [Hz]	Pixel rate [kHz]	Notes
	Our system	Chirped longitudinal AOD with parallel detection	66×14	10 ns	0.59 μs / frame	800,000	739,200	Oversampling our resolvable points.
3	Nadella, 2016	3D random access and continuous line scanning with shear AOD	51×50	50 ns	24.5 μs / line	739	1,885	
			512×512	50 ns	24.5 μs / line	39	10,200	
			1024×1024	50 ns	24.5 μs / line	12.9	13,500	
4	Fernandez-Alfonso, 2014	Shear AOD/AOL for random access imaging	512×512	4 μs	24 μs / point	0.14	36	
			200×200	4 μs	24 μs / point	0.89	36	
5	Chen, 2011	Chirped shear AOD	250×80	50 ns		1,000	20,000	
			500×500	50 ns		80	20,000	
6	Grewe, 2010	Random access shear AOD	256×256	2 μs	10 μs / point	1.27	83	
7	Roorda, 2004	Longitudinal chirped AOD	512×480	100ns	2.77 μs / line (estimated)	30	7,400	Oversampling resolvable points
1	Bouchard, 2015	Camera-based light sheet microscopy with polygonal scanner (SCAPE)	1400×80			1,000	40,000	
			400×40			2,404	38,464	
			800×80			2,404	153,856	
8	Cheng, 2011	16 kHz resonant mirror, 4 beam multiplexing	512×512			250	65,536	
9	Duocastella, 2017	Camera-based light sheet microscopy with TAG lens	512×512			330	86,508	
10	Chen, 2014	Camera-based light sheet microscopy with Bessel beam	512×512			333	87,294	
2	Mikami, 2018	Frequency multiplexing with an AOD	190×231			16,000	702,240	
	Andor Neo 5.5 sCMOS	Commercial sCMOS (~200 kHz line rate)	2048×8-2560×2160			27,057-100	552,960	

Supplementary Table 1 | Summary of imaging speeds for current 3D, biological fluorescence imaging technologies.

	Manufacturer	Product Number	Bandwidth [MHz]	Aperture size [mm]	Acoustic velocity [mm μs^{-1}]	Max N	Scan period [μs]	N	N/s [MHz]	Scan frequency [MHz]
1	Gooch and Housego	3200-120	75	2.5	4.26	44	1.25	23	19	0.80
2	Gooch and Housego	4245-121	150	3.2	4.26	113	1.50	56	38	0.67
3	Gooch and Housego	4245-121	150	5	4.26	176	2.35	88	38	0.43
4	Gooch and Housego	4200-VI	150	5	0.71	1056	14.08	528	38	0.07

Supplementary Table 2 | Currently available AODs and suggested operating parameters to reach a maximum rate of resolvable points. The listed bandwidths are 1.5× the manufacturer's listed bandwidth, as used in the paper. The aperture sizes are extended by removing the AOD cover. AOD #1 is the AOD used in the current manuscript. AOD #2-4 are used in Fig. 5 and Supplementary Fig. 14.

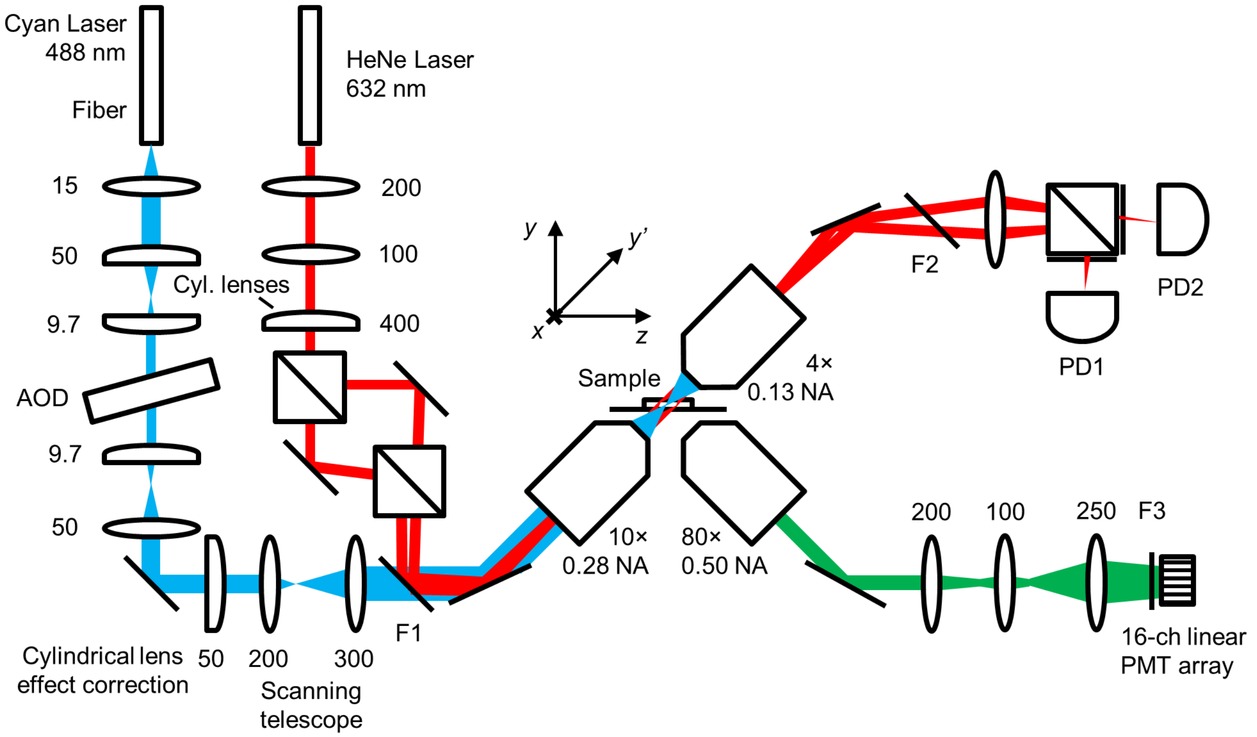

Supplementary Figure 2 | Detailed optical setup. All focal lengths are given in mm next to individual lenses. A cyan excitation laser is collimated by an aspheric lens and shaped by a cylindrical telescope to fill the 2.5×0.45 mm^2 aperture of the longitudinal TeO_2 AOD. In the non-scan direction, the effect of the cylindrical telescope is reversed by a cylindrical lens and spherical lens. In the scanning direction, the 50-mm spherical lens and a 50-mm cylindrical lens correct the cylindrical lens effect caused by driving the AOD by a chirped signal to obtain the tightest focus at the sample. A scanning telescope underfills the scanning beam to the back aperture of the excitation objective. At the sample, the beam excites a ~70 μm line in the y'-direction, angled 45° with respect to the y-direction, and scans a 60 μm FOV in the x-direction. The excited plane is imaged by an orthogonal collection objective to 14 channels of a 16-channel linear PMT array after filtering by a bandpass filter (F3). The presence and speed of individual animals are detected using a second setup. A HeNe laser is shaped and split into two beams with angular offsets, and diverted towards the excitation objective by a low-pass beamsplitter (F1), forming two 5 ± 1 μm thin light sheets separated by 210 ± 1 μm at the sample. The two beams are trans-collected, filtered by a high-pass beamsplitter (F2), and detected individually by two iris and photodiode pairs (PD1, PD2).

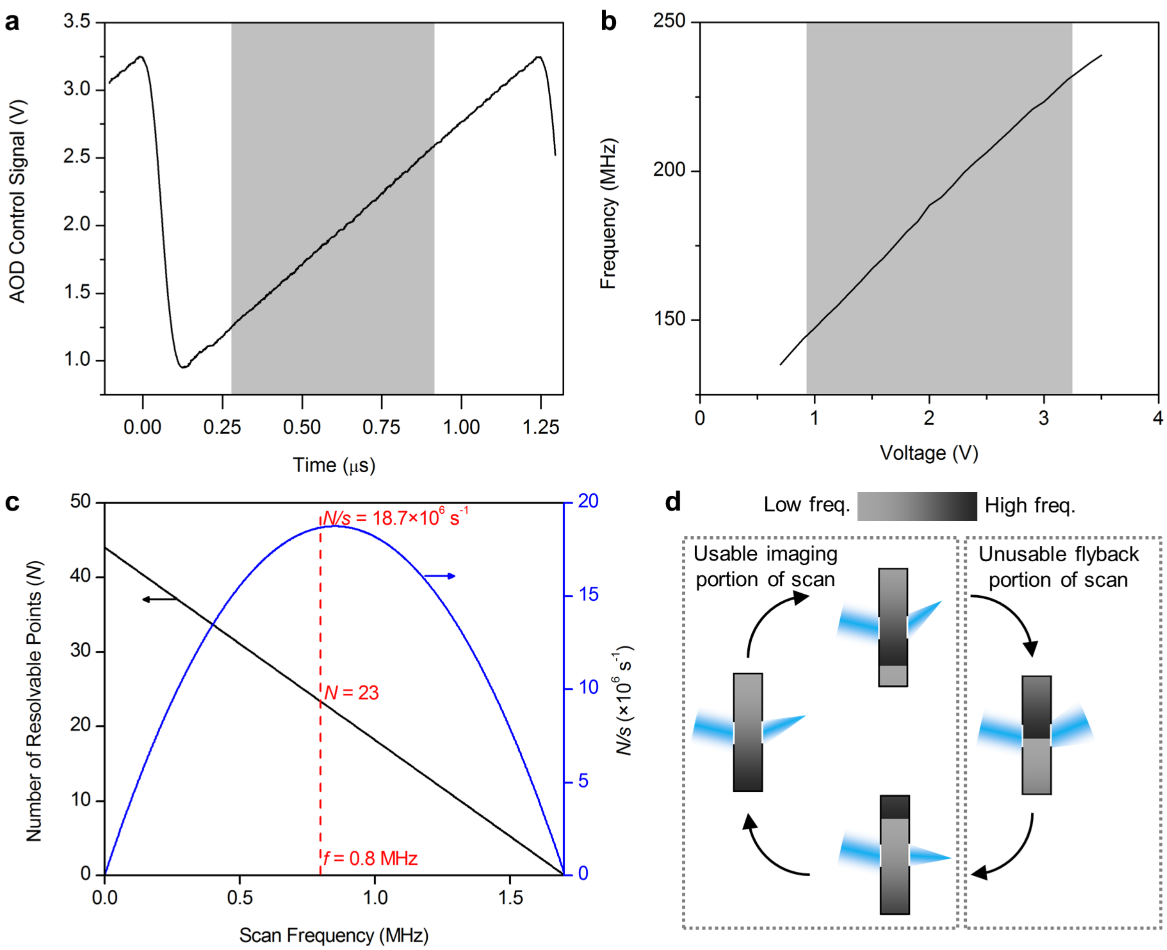

Supplementary Figure 3 | Characterization of the scanning properties of the acousto-optic deflector (AOD). (**a**) The function generator signal with $V_0 = 2.1$ V, $\Delta V = 2.3$ V, and $t = 1.25$ μs (800 kHz). The region of the scan used for imaging is highlighted in gray. (**b**) The AOD driver converts the function generator signal into a linear frequency chirp that propagates in the AOD crystal. The AOD driver displays linear frequency-voltage response beyond its rated $f_0 = 200$ MHz and $\Delta f = 50$ MHz. We use $\Delta f = 75$ MHz to generate more resolvable spots in the AOD. (**c**) Our AOD driven with a chirped frequency theoretically has a reduction in the number of resolvable points as scan frequency increases. The number of resolvable points per second reaches a maximum when the scan period is half the response time of the AOD, i.e. when the beam is scanned back and forth as quickly as possible. The red line indicates our operating point. (**d**) The AOD is driven by the chirped frequency waveform presented in (a) to deflect the beam. In the schematic, the frequencies are represented by the grey-scale gradient. The scanning displays two regimes: the usable regime when the upchirped portion of the acoustic wave propagates across the AOD aperture that continuously deflects the beam as desired at the sample, and the unusable regime when the downchirped portion of the signal crosses the aperture, causing different portions of the beam to deflect at different angles.

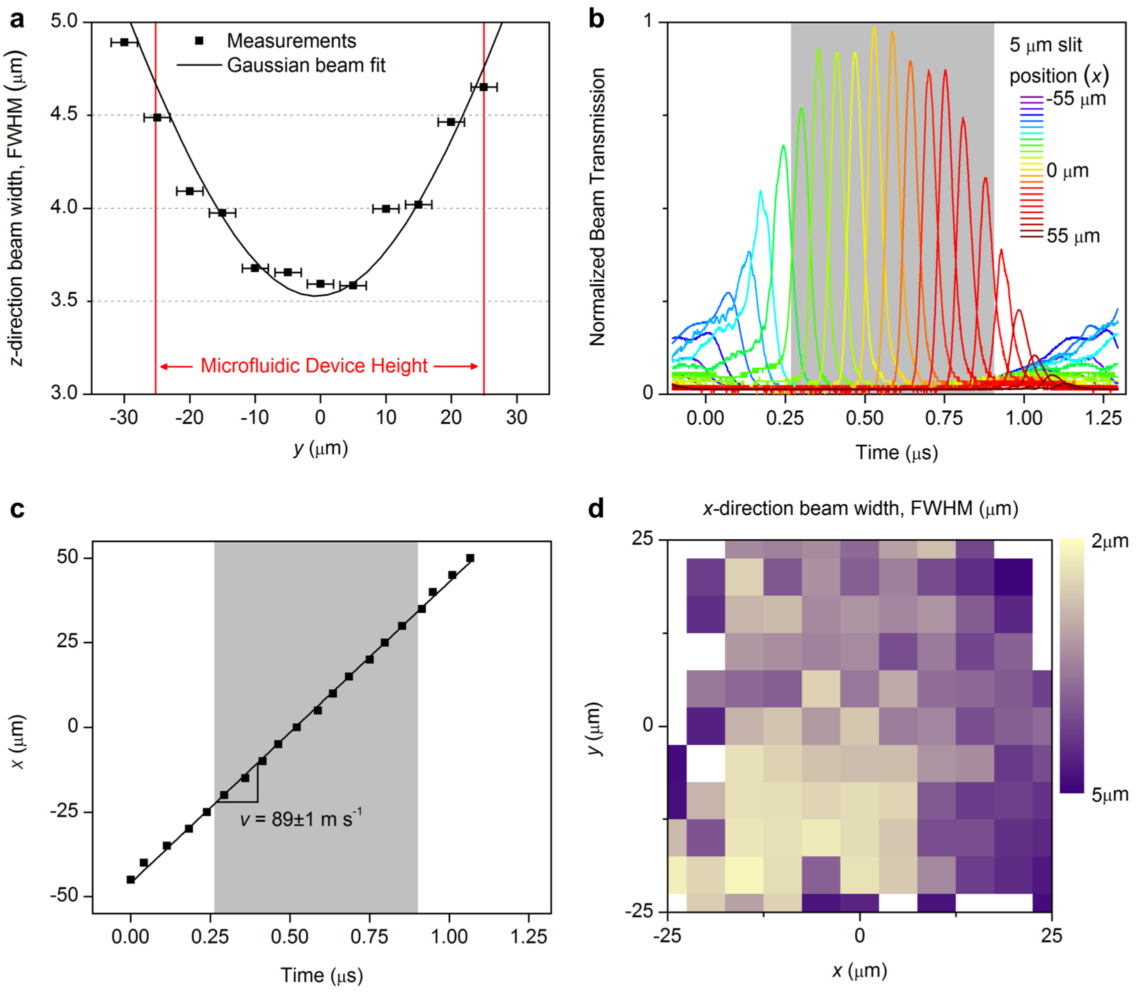

Supplementary Figure 4 | Laser beam profile characterization in free space, without the microfluidic device. (**a**) The beam size in the flow-direction measured by the knife edge test follows Gaussian beam propagation, with a FWHM between 3.5 – 4.5 μm where the imaging channel is located. Error bars represent uncertainty in the position of the knife edge. (**b**) To determine the beam FWHM in the scan-direction, we scanned the beam across a 5 μm wide slit and measured transmission. The slit is translated in 5 μm increments in the scan direction from -55 μm to +55 μm. The usable imaging period is highlighted in gray. Here, we show the slit positioned at the focal plane (z = 0). The experiment is repeated for a range of y positions (c). (**a**) The position and velocity of the peak intensity of the beam in (a) as a function of time. The speed is 89 ± 1 m s^{-1} and is linear in the imaging region. (**d**) The beam FWHM in the scan direction as a function of x and y, found by deconvolving transmission profiles (such as in (a)) by a 5 μm slit. The imaging channel of the microfluidic device is within a region where the beam FWHM is at a minimum.

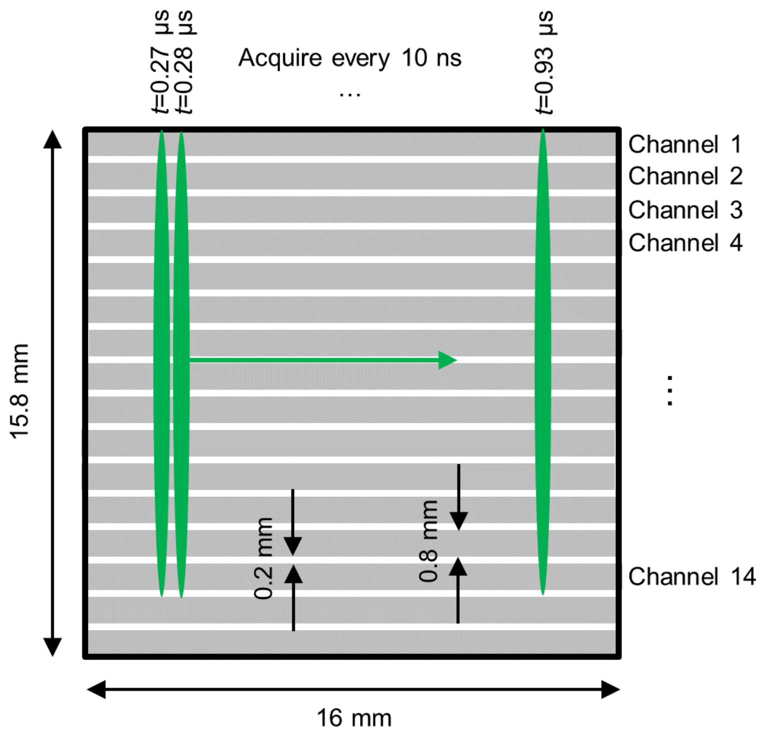

Supplementary Figure 5 | PMT and imaging details. The full excitation field-of-view of 60×70 µm^2 (x-y') is imaged onto the PMT array with overall magnification of 200×. Each element with height 0.8 mm images a 4 µm axial slice (y') of the scanning beam. The imaging resolution in the y'-direction is defined by the demagnified PMT element separation of 5 µm (3.5 µm in the y-direction). The 0.2 mm dead space between PMT elements results in some signal and information loss. As the beam scans, the detected light hits different portions of the PMT. Data is acquired every 10 ns. During the usable portion of the scan when the excitation beam is focused on the sample (from 0.27 µs to 0.93 µs into each scan), collected data is used towards forming images (see Supplementary Figure 3). During the unusable portion of the scan, when the excitation beam is not tightly focused or distorted at the sample, collected data is unused.

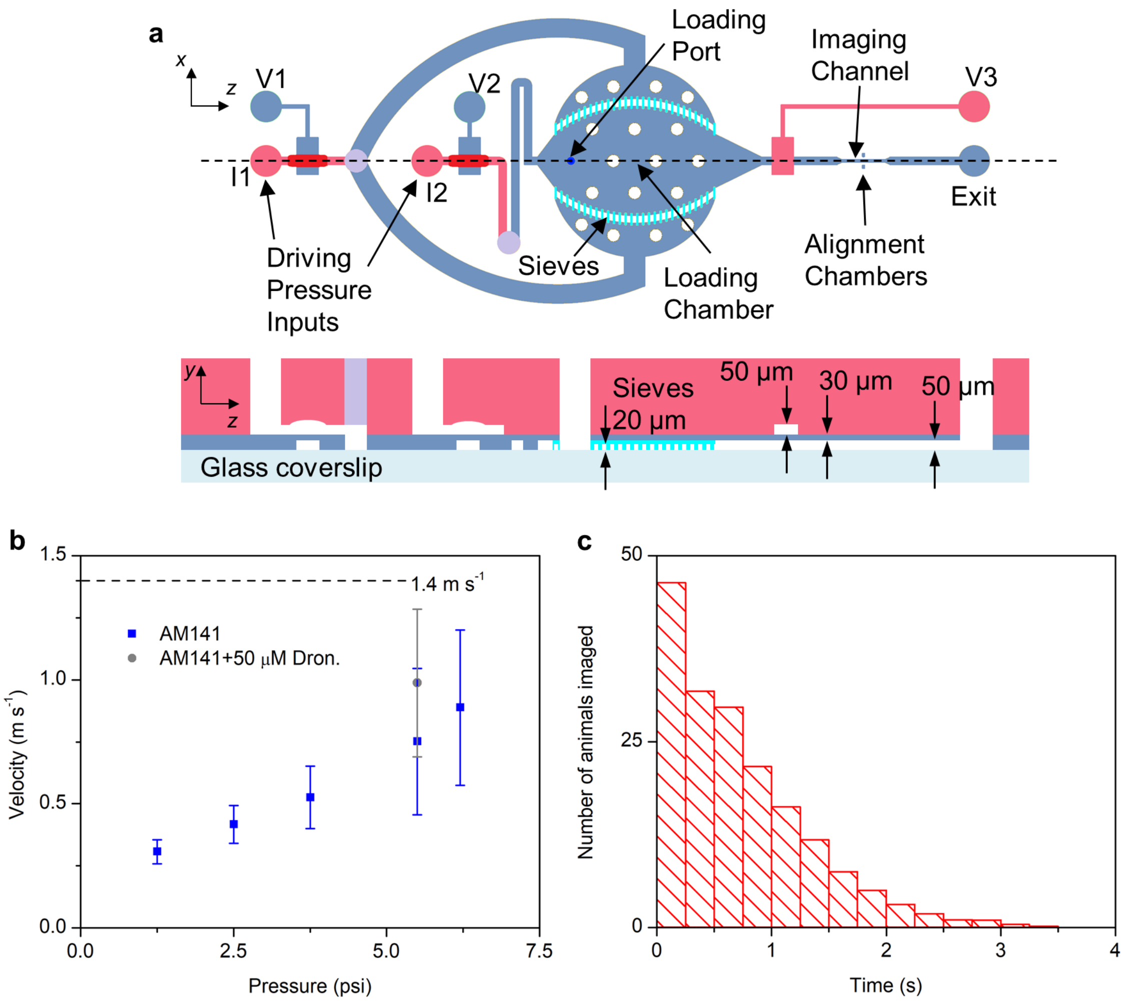

Supplementary Figure 6 | Microfluidic device details and flow characteristics. (**a**)Microfluidic device schematic & cross-section. Hundreds of animals are loaded into the main chamber through the loading port, and the sieve structure prevents backflow of the worms through the inputs. The input ports (I1,I2) apply a driving pressure to the animals to send them through the imaging channel, and the valves (V1,2,3) control when that pressure is applied. Fluorescent beads loaded into the alignment chambers on both sides of the imaging channel aid in device alignment with the excitation beam. (**b**) Animal velocity vs driving pressure at I1 and I2. For polyQ40 animals without drugs and those treated with 25 μM dronedarone, 6.2 psi is used to flow animals at 0.89 ± 0.31 m s^{-1} speed limit. Animals treated with 50 μM dronedarone tend to be smaller, and only 5.5 psi is required for flow at 0.99 ± 0.30 m s^{-1}. (**c**) Animal frequency distribution vs time for the average experiment with a population of ~175 animals. The majority of animals are imaged in the first second of imaging because the rate of animals moving through the channel depends on the concentration of animals in the loading chamber.

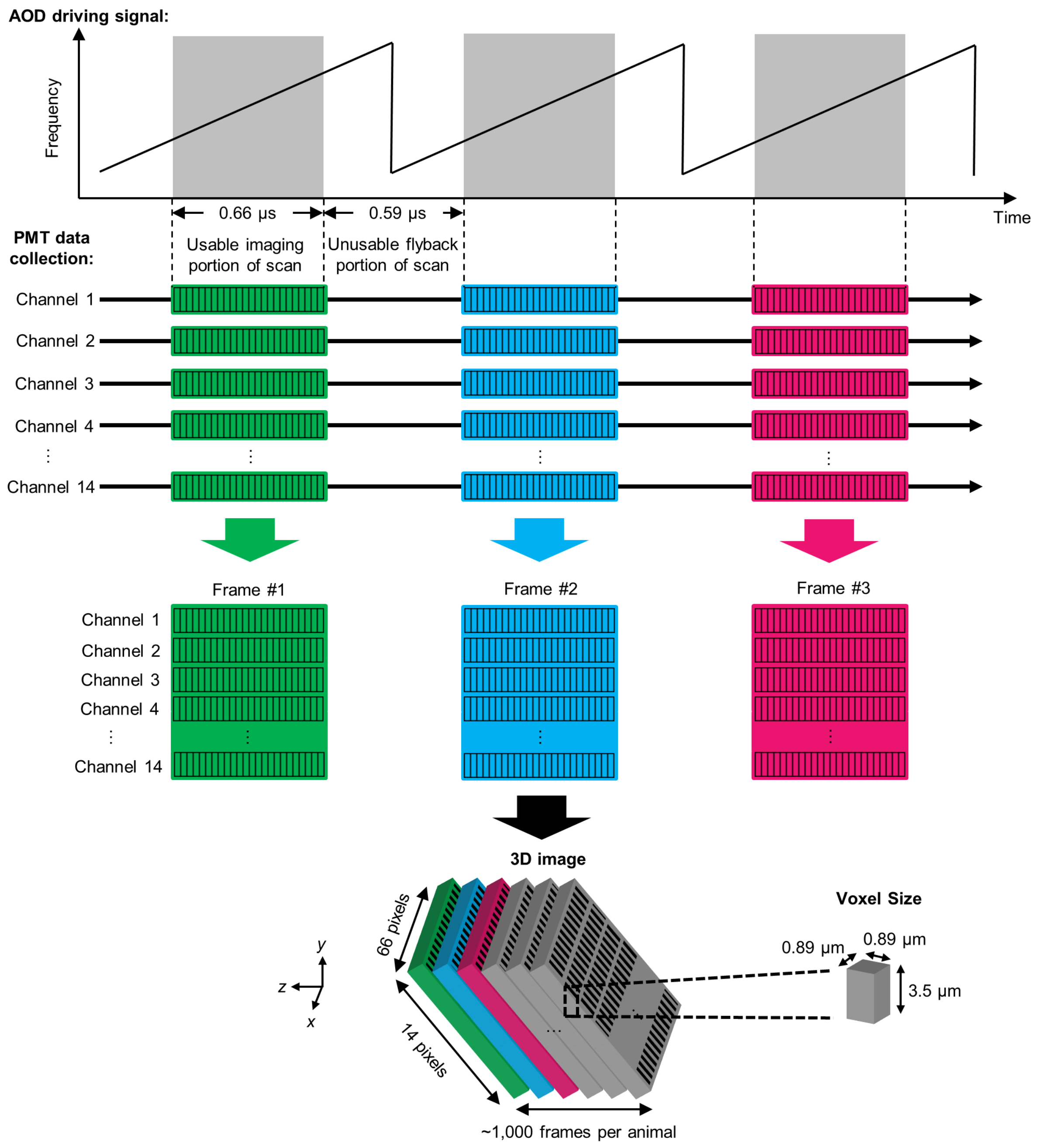

Supplementary Figure 7 | Frame acquisition and image formation from PMT data. Data collected during the usable, 0.66 μs long portion of the scan (highlighted gray region in the frequency-time plot) is used to form frames. Each frame consists of 66 data points captured every 10 ns by each of the 14 PMT elements to give frames with 66×14 (x-y') pixels. The frames are captured every 1.25 μs. To form the 3D image, the frames are stacked, and then skewed to account for the geometry of the system – the animal flow-direction (z) is at ~45° with respect to the excitation and imaging plane (x-y'). The pixels are converted from the skewed coordinate system (x-y'-z) to Cartesian coordinates (x-y-z) by compressing the pixel in the y-direction. After the compression and velocity correction, each pixel has dimensions 0.89×3.5×0.89 μm^3 (x-y-z).

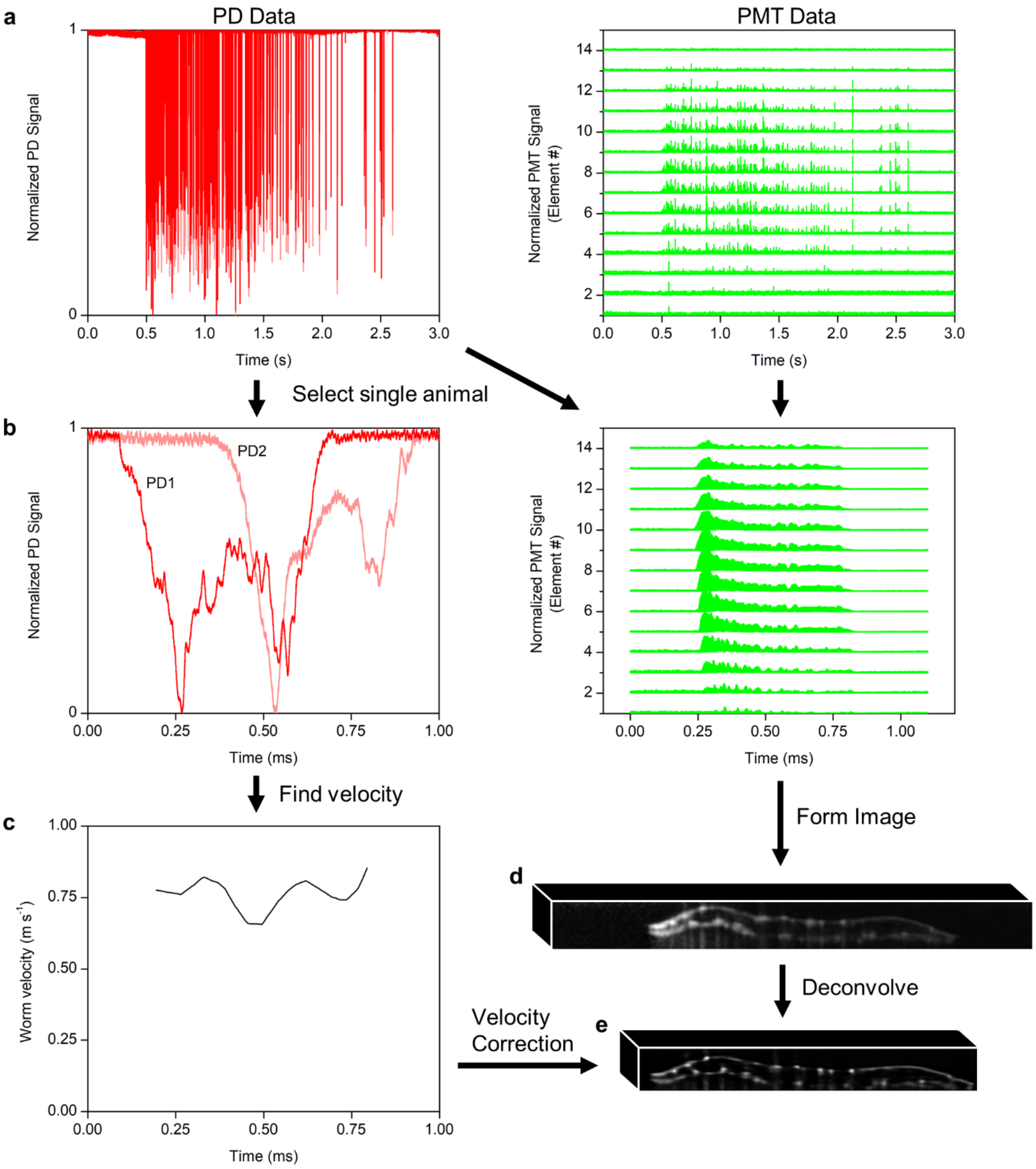

Supplementary Figure 8 | Overview of the image formation algorithm. (**a**) Two photodiodes record the transmission of two HeNe beams for animal speed detection. Periods of low transmission in the PD data indicates when a *C. elegans* animal is in the imaging region. The 14 PMT channels record fluorescence signal from the animals. (**b**) Single animals are cropped out of the full PD and PMT data. (**c**) A dynamic time warping algorithm uses the two PD transmission signals to find the point-by-point time delay and velocity of the animal moving through the imaging channel. (**d**) A volumetric image of the animal is formed as described in **Supplementary Fig. 7**. (**e**) A final 3D image is formed using the velocity data to resize the pixels to 0.89×0.89 μm^2 (x-z), and by 3D deconvolution using the average fluorescent bead PSF. During velocity correction, a minimal amount of interpolation occurs.

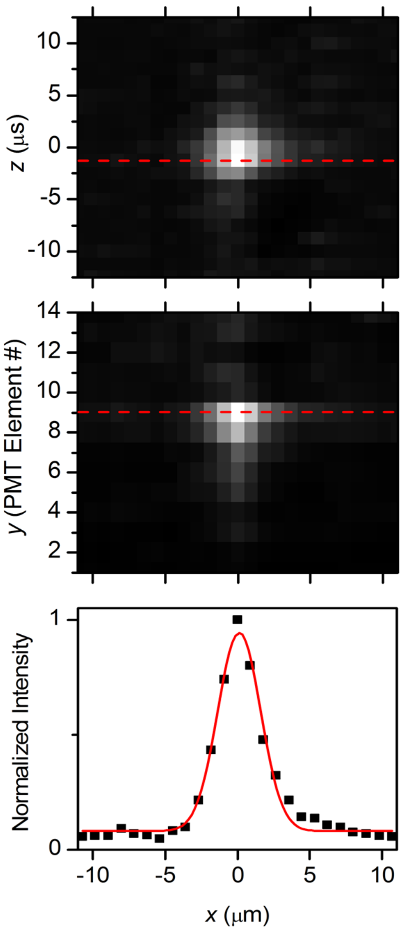

Supplementary Figure 9 | Example image of a 0.5 μm bead flowing at ~1 m s^{-1} in the microfluidic device, with the scan-direction (x) FWHM = 3.4 ± 0.1 μm. The bead displays aberrations in the y-direction due to the tilted device.

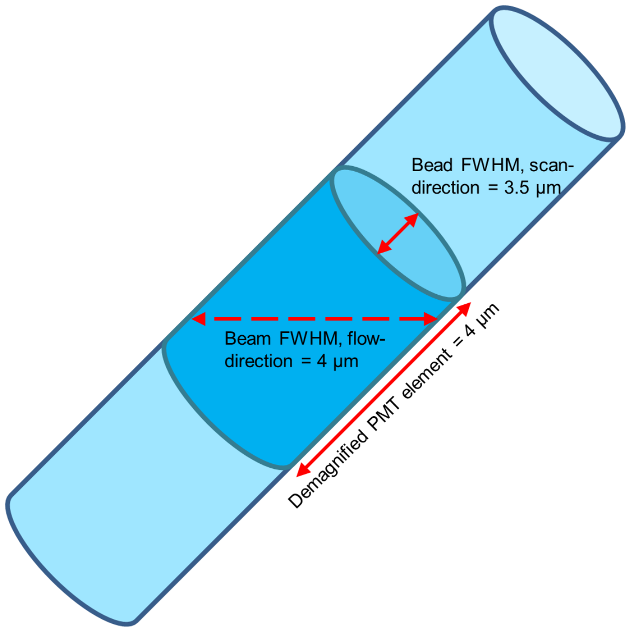

$$V = \pi \frac{\mathrm{FWHM}_x}{2} \frac{\mathrm{FWHM}_z}{2\sqrt{2}} \Delta \mathrm{y}'$$
$$= \pi \frac{3.5\ \mu\mathrm{m}}{2} \frac{4\ \mu\mathrm{m}}{2\sqrt{2}} 4\ \mu m$$
$$= 31\ \mu\mathrm{m}^3$$

Supplementary Figure 10 | The average volume imaged by a PMT element. The radii of the cylinder are determined by the average bead PSF in the flow-direction (z) and the scan-direction (x). The height of the cylinder is the demagnified PMT element size.

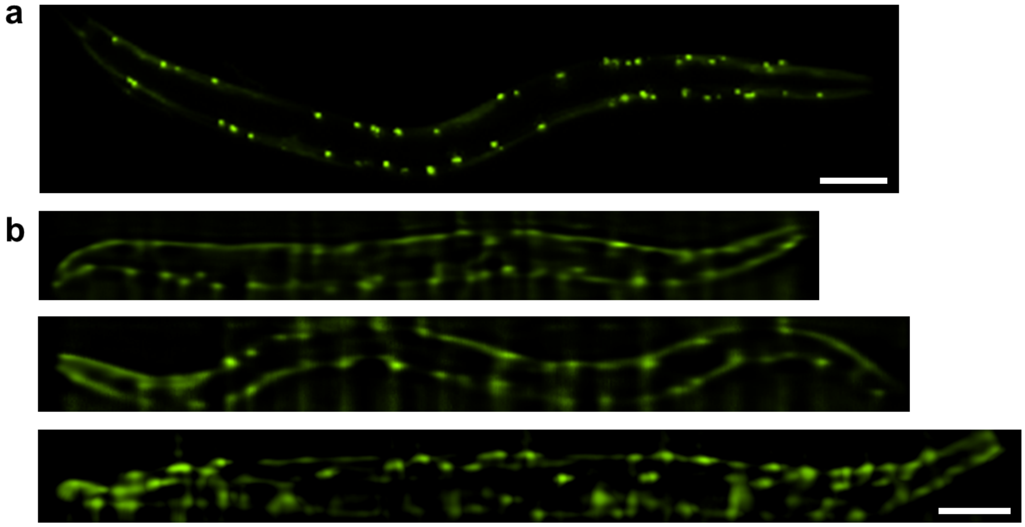

Supplementary Figure 11 | Comparison of *C. elegans* images obtained with line-scanning fluorescence microscopy and wide-field microscopy. (**a**) PolyQ40 strain animal imaged with an inverted microscope and 4×, 0.13 NA objective and 84 ms exposure time. Scale bar = 50 µm. (**b**) Multiple examples of PolyQ40 strain animals imaged with our platform (maximum intensity projection) within 1 ms. The first animal is in the early L4 stage, the second in mid L4 stage, and the third in the late L4 stage. The number of aggregates increases with age, with the late L4 stage having similar numbers to (a). Scale bar = 50 µm.

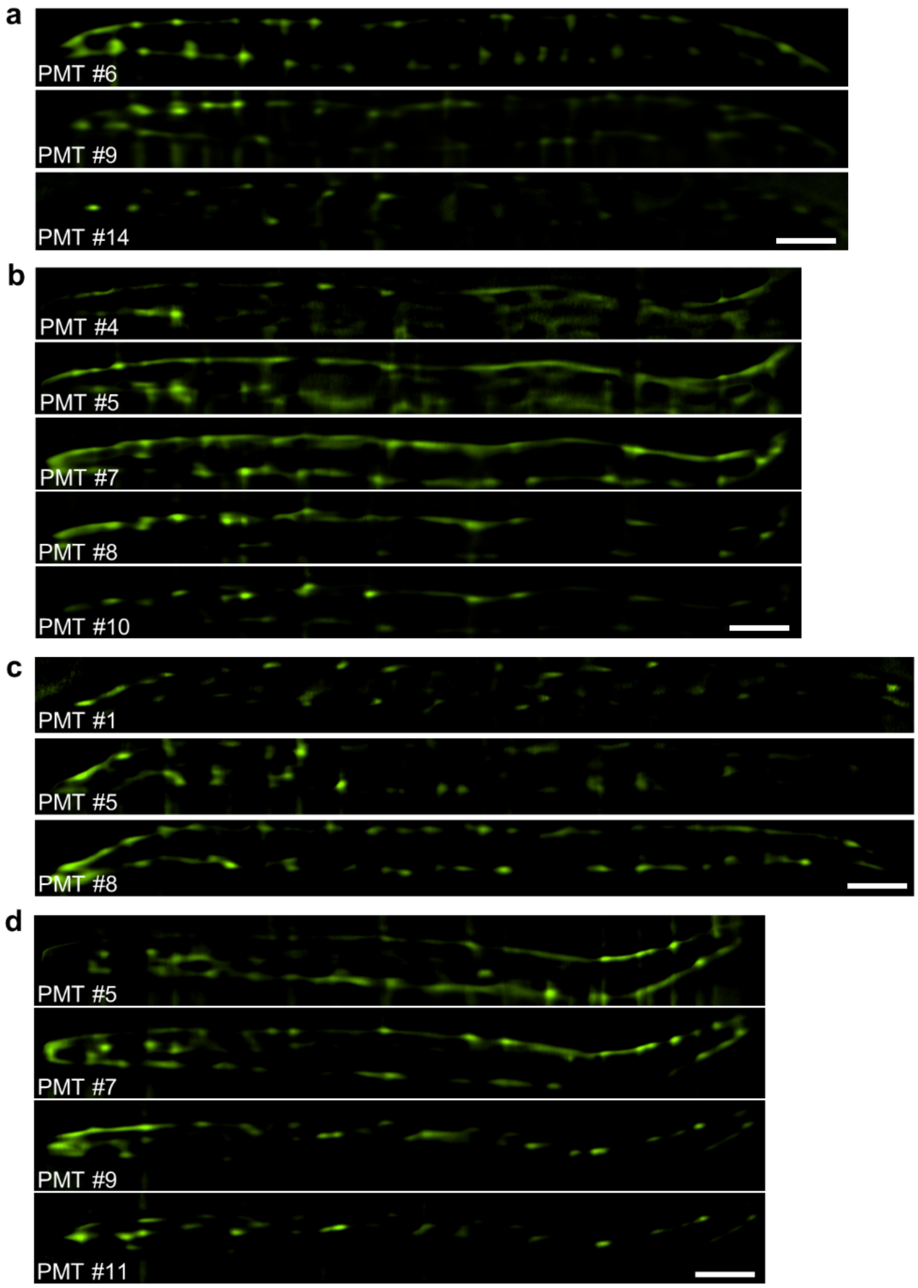

Supplementary Figure 12 | (**a-d**) Four different polyQ40 animals, each showing planes captured by different PMT elements. In some animals, aggregates or diffused fluorescence are stacked across multiple planes, showing the need for 3D imaging to resolve each aggregate. Scale bars = 50 μm.

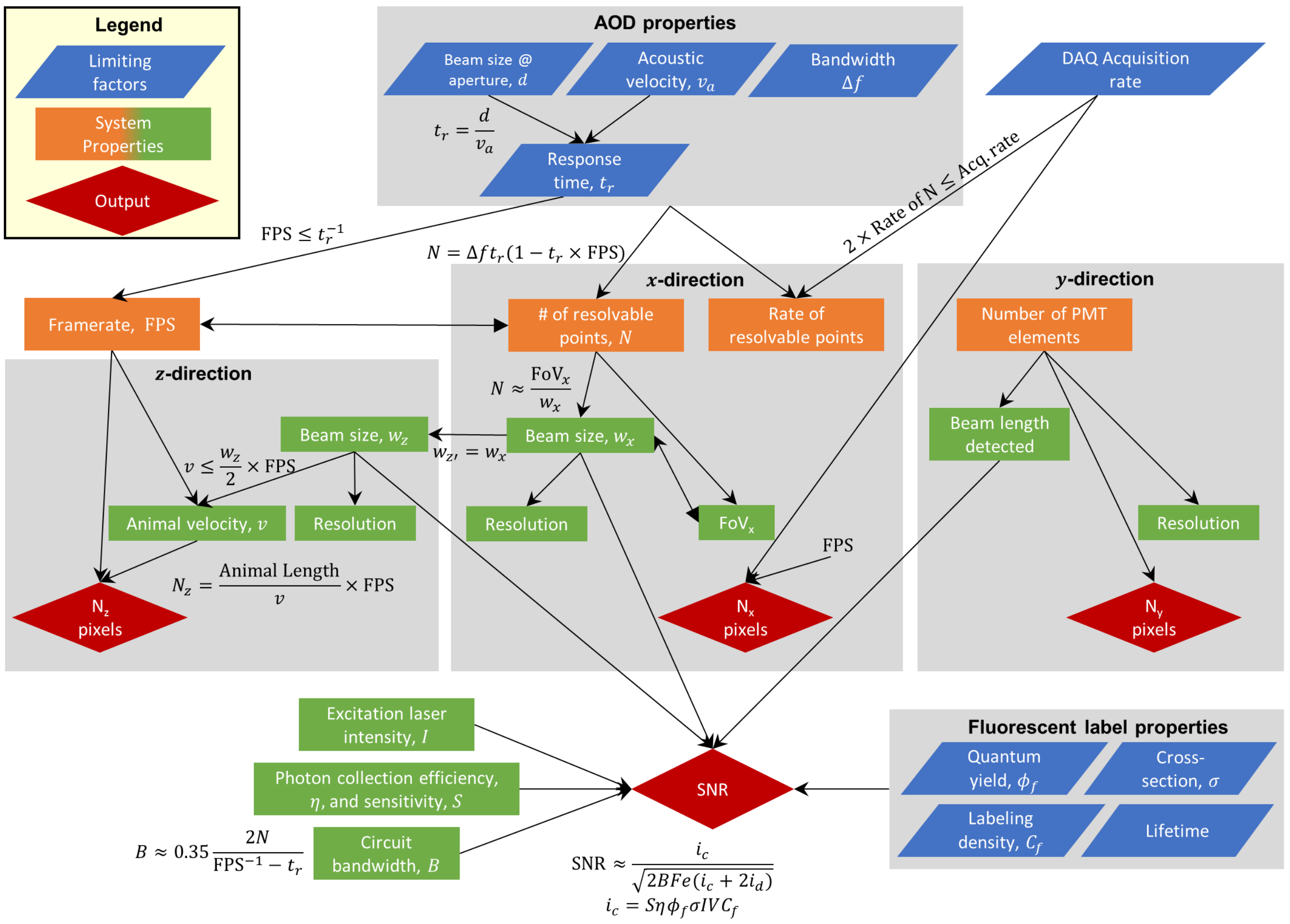

Supplementary Figure 13 | LEAD microscope design considerations. The primary factors defining the performance and limitations of the system are the AOD properties, data acquisition rate, and fluorescent label properties. Intermediate system properties include the framerate, number of resolvable points from the AOD, rate of resolvable points from the AOD, and the number of PMT elements used for imaging. More practical system properties include beam sizes, resolution, and field-of-view. Finally, the final image is defined by the number of pixels and the signal-to-noise ratio.

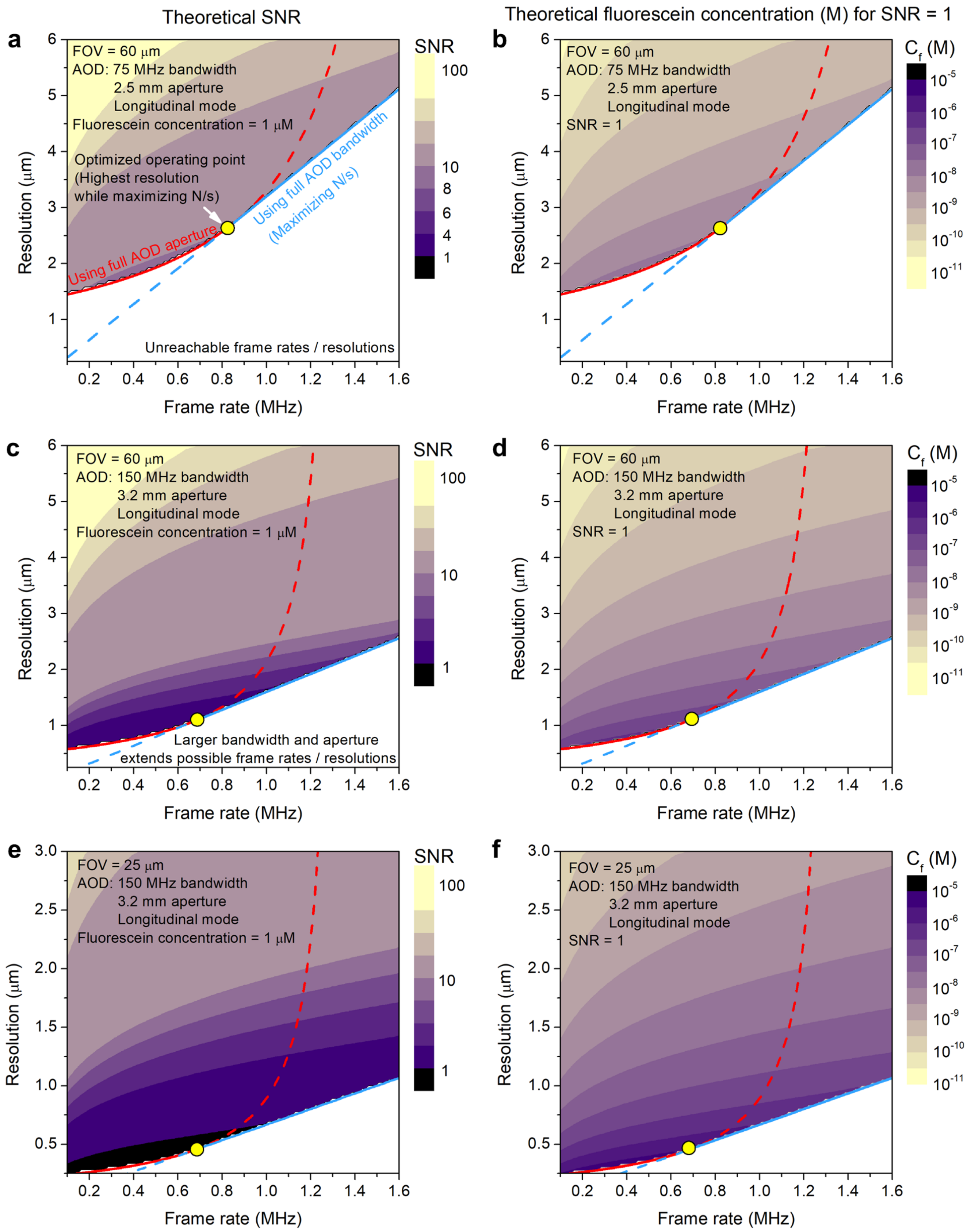
Theoretical SNR
Theoretical fluorescein concentration (M) for SNR = 1
a
FOV = 60 μm
AOD: 75 MHz bandwidth
2.5 mm aperture
Longitudinal mode
Fluorescein concentration = 1 μM
Optimized operating point
(Highest resolution
while maximizing N/s)
Using full AOD aperture
Using full AOD bandwidth
(Maximizing N/s)
Unreachable frame rates / resolutions
SNR
b
SNR = 1
C_f (M)
c
AOD: 150 MHz bandwidth
3.2 mm aperture
Larger bandwidth and aperture
extends possible frame rates / resolutions
d
e
FOV = 25 μm
f
Resolution (μm)
Frame rate (MHz)

Supplementary Figure 14 | Theoretical signal-to-noise ratios and detection limits for our LEAD system. (**a**) The theoretical SNR of LEAD as a function of frame rate and resolution, for a FOV of 60 μm, fluorescein concentration of 1 μM (typical for cells[11]), and using the current AOD (75 MHz bandwidth, 2.5 mm aperture, longitudinal TeO_2). Some resolution and frame rate combinations are unreachable, limited by the AOD aperture or bandwidth (white region). For the highest resolution imaging, the full AOD aperture is used. For high-speed imaging, it is advantageous to underfill the AOD aperture to utilize the full AOD bandwidth and reach the maximum rate of resolvable points during the scan period. We operate our system at the optimal point where the highest resolution is reached while maximizing the rate of resolvable points (yellow dot). (**b**) The minimum fluorescein concentration for SNR = 1 for the system in (a). (**c**) Theoretical SNR for a FOV of 60 μm and fluorescein concentration of 1 μM using a higher bandwidth, larger aperture AOD (150 MHz bandwidth, 3.2 mm aperture, longitudinal TeO_2, Supplementary Table 2). The larger aperture and higher bandwidth extend the range of resolution and frame rate combinations. Furthermore, higher SNR is reached for identical imaging conditions because the same number of resolvable points can be reached with a lower rate of resolvable points during the imaging portion of the scan, allowing lower bandwidth circuits with less noise. (**d**) The minimum fluorescein concentration for SNR = 1 for the system in (c). (**e**) Theoretical SNR for a LEAD system imaging a smaller FOV of 25 μm (for applications such as cellular imaging), and using the AOD in (c-d). The black region indicates the regime where SNR < 1 for 1 μM fluorescein. (**f**) The minimum fluorescein concentration for SNR = 1 for the system in (e). See Supplementary Note 4 for details on assumptions made for the figures.

Supplementary Note 1: Calculating the collection efficiency, detection limit, and SNR.

The excess noise factor from cascaded electron multiplication is calculated by: $F = g/(g-1)$, where g is the gain of each dynode[12]. Our PMT has 10 dynodes, and we use an overall gain of 10^6, resulting in g = 4 ($g^{10} = 10^6$), resulting in $F = 1.33$. The cathode-equivalent dark current is the anode dark current (0.01 nA) divided by the overall gain (10^6), resulting in $i_d = 0.01$ fA.

The power of fluorescence photons incident on each PMT element is $P = \eta\phi_f\sigma IVC_f$, where η is the collection efficiency of the optics, $\phi_f = 0.93$ is the fluorescence quantum yield of fluorescein, $\sigma = 2.92\times10^{-16}$ cm^2 is the absorption cross-section of fluorescein[13], $I = 0.51$ GW m^{-2} is the illumination intensity for the available laser power ($P = 4$ mW), $V = 31$ μm^3 is the average volume detected by each PMT element, and C_f is the concentration of fluorescein. The cathode current of each PMT element is $i_c = SP$, where S = 50 mA W^{-1} is the radiant sensitivity of the PMT. Substituting $i_c = SP$ in $\mathrm{SNR} = i_c/\sqrt{2BFe\left(i_c + 2i_d\right)}$ and fitting the SNR data, we obtain the collection efficiency of the optics $\eta = 2.9\%$ and detection limit of $C_f = 22.3$ nM.

The photon power being detected by each PMT element is $P_d = QP$, where $Q = 0.15$ is the quantum efficiency of the PMT. At the detection limit, we obtain $P_d = 6.2\times10^7$ photons s^{-1}. The integration time of the detection system is characterized by the circuit bandwidth: $\sim 0.35/B = 17.5$ ns. During this integration time, each PMT element detects 1 photon at the detection limit. The cathode current at the detection limit is ~8 pA, much larger than the cathode-equivalent dark current of 0.01 fA. The negligible dark current indicates that our LEAD imaging system is shot-noise limited.

Supplementary Note 2: The saturation intensity of fluorescein is approximated by:

$$I_s \approx \frac{1}{\sigma\tau}$$

Where σ = 2.92×10^{-20} m^2 is the absorption cross-section of fluorescein, and τ = 4.1 ns is the lifetime of fluorescein, giving I_s = 340 kW cm^{-2} or P_s = 27 mW with our average excitation cross-section.

The detection limit of the system, in terms of number of molecules, is calculated from the measured detection limit (concentration) and the average PSF volume:

$$N = 22.4 \text{ nM fluorescein}\cdot\frac{N_A \text{ molecules}}{1 \text{ mole}}\cdot\frac{1000 \text{ L}}{1 \text{ m}^3}\cdot\frac{31\times10^{-18} \text{ m}^3}{1 \text{ PSF volume}} \approx 420 \text{ molecules fluorescein}$$

At saturation intensity, ~7× higher than the intensity used in this study, the detection limit is expected to decrease to 60 molecules of fluorescein. The detection limit would further decrease if imaging speed is decreased.

Supplementary Note 3: Limitations of LEAD microscopy.

Factors limiting resolution and field-of-view:

- **Number of resolvable spots from AOD (*N*).** In the scan-direction (x), the resolution and field-of-view are defined by the number of resolvable spots from the AOD. The number of resolvable spots is the ratio of maximum scan angle of the AOD and the divergence of the beam at the AOD. AODs have a lower angular scan range compared to traditional galvanometric scanners, and thus fewer resolvable spots (galvanometric mirrors have thousands of resolvable spots)[14]. However, more resolvable spots can be obtained by slower scanning, an AOD with higher bandwidth, or an AOD with a larger aperture or in shear configuration (at the cost of frame rate). For example, the system could have improved resolution by using AOD #2 (Fig. 5, Supplementary Table 2), which has recently become available and has higher bandwidth than our AOD. AOD #2 driven at 0.8 MHz could result in 45 resolvable points, improving our resolution 2×. A shear AOD driven slowly can provide over 1,000 resolvable points (AOD #4, Supplementary Table 2), approaching mirrors.
- **Number of detector elements.** In the imaging-direction (y'), the resolution and/or field-of-view can be increased with more detector elements. Currently, PMTs are available with 32 elements, and silicon photomultipliers can be configured for even more elements. However, acquisition of data is limited by current data acquisition. DAQs with 16 channels at 100 MHz have become available only recently. For more detector elements, multiple DAQs and hard drives would have to be used in parallel.

Factors limiting signal-to-noise ratio:

- **Excitation volume.** As excitation volume goes up, more fluorophores are available to absorb and emit light, increasing SNR.
- **Available laser power.** Our laser was limited to delivering just 4 mW to the sample, when ~27 mW would be close to saturation.
- **Imaging speed.** As the scanning speed, rate of resolvable points from the AOD (*N/s*), and data acquisition rate increase, the number of photons collected from a point on the sample decreases. The designed PMT circuit bandwidth should also scale with the rate of resolvable points during the usable scan period to keep up with the fast modulation of the signal from the sample, which will introduce more noise.
- **Photon collection efficiency.** The collection efficiency of our optics is 2.9 ± 1.2% and can be increased with a higher numerical aperture collection lens. However, our system requires long working distance objectives to prevent contact with the microfluidic device. The objective we used is one of the highest numerical aperture objectives with the required working distance.
- **Fluorophore properties.** Brighter fluorophores with higher saturation intensities and a higher labeling density would contribute towards higher SNR in our *C. elegans* images.

Factors limiting imaging speed:

- **Signal-to-noise ratio.** Ultimately, the imaging speed depends on the number of photons that can be collected from the sample. See Supplementary Figure 14.
- **Frame rate.** The ideal and maximum framerates are both limited by the response time of the AOD, which depends on the AOD aperture size (and beam size entering the aperture) and the acoustic velocity of the AOD crystal (see Methods). We have used an AOD with a small aperture (2.5 mm), and the fastest crystal (longitudinal TeO_2 with v_a= 4,260 m s^{-1}) for 488 nm light. The beam size can be reduced below the aperture size to increase the framerate further, at the cost of number of resolvable points (resolution and FOV). The ideal frame rate for our AOD, giving the most resolvable points per second (*N/s*), is 0.852 MHz. We used 0.8 MHz because this is the maximum frequency allowed by the bandwidth of our function generator. Increasing the framerate

beyond 0.852 MHz results in a lower number of resolvable points per second, and even fewer resolvable points per scan, but may be useful for applications where high framerates are absolutely necessary. For example, driving AOD #2 (which has an optimal frequency of 0.67 MHz) at 0.8 MHz can potentially increase our system's resolution, as described above.

- **Pixel rate.** The data acquisition rate should be at least twice the rate of resolvable points from the AOD during the scan period. The peak *N/s* is limited by the AOD bandwidth (maximum *N/s* = bandwidth/4). AODs with higher bandwidths may become available in the future. The pixel rate is also limited by the data acquisition card (ours has a maximum of 100 MHz). Single channel data acquisition cards with higher sampling rates are available, but are generally limited by the speed of analog-to-digital conversion.

Factors limiting overall animal throughput:

- **Signal-to-noise ratio.** Ultimately, the imaging speed depends on the number of photons that can be collected from the sample. See Supplementary Figure 14.
- **Animal velocity.** The maximum animal velocity is limited by the size of the beam in the flow direction and the framerate of the system. The animal velocity cannot exceed $v = \mathrm{FWHM}_z / 2 \times \mathrm{Framerate}$ for proper sampling.
- **Animal deli[13]very.** Our current microfluidic device can hold and image up to 300 animals from a single population at a time. For continuous animal delivery, and delivery of distinct populations, our device can potentially be connected to our previously developed multiplexed delivery device[15]. The multiwell device can be loaded with different populations of *C. elegans* using widely available liquid handling systems to deliver a single population made of hundreds of animals to our imaging system in under 3 seconds per population.

Supplementary Note 4

For Fig. 5 and Supplementary Fig. 14, we use the PMT shot-noise model, $\mathrm{SNR} = i_c \big/ \sqrt{2BFe(i_c + 2i_d)}$ and $i_c = S\eta\phi_f\sigma IVC_f$, where $S = 50$ mA W^{-1} is the radiant sensitivity of the PMT, $\eta = 2.9\%$ is the collection efficiency of the optics, $\phi_f = 0.93$ is the fluorescence quantum yield of fluorescein, and $\sigma = 2.92 \times 10^{-16}$ cm^2 is the absorption cross-section of fluorescein. For the other parameters, we assume an ideal system:

1) We use the saturation intensity of I_s = 340 kW cm^{-2} to maximize SNR (our current setup uses ~50 kW cm^{-2}).
2) The number of PMT elements scales such that the demagnified element size equals the resolution.
3) The desired FOV and resolution determines the number of resolvable points from the AOD: $N = \mathrm{FOV} / \mathrm{resolution}$.
4) The beam size entering the AOD aperture, d, is selected to meet the desired N and frame rate, while minimizing the rate of resolvable points during the usable imaging period (and minimizing the circuit bandwidth and noise), according to $N = \Delta f\, d/v_a \left(1 - d/v_a t\right)$, where $t = (\text{Frame rate})^{-1}$.

5) The bandwidth, B, of the PMT circuit is set according to the rate of resolvable points from the AOD during the usable imaging period: $\text{Rise \& Fall Time} = \left(t - d/v_a\right)/2N$, and $\text{B} = 0.35/(\text{Rise \& Fall Time})$.
6) We assume the data acquisition card can acquire at 1/(Rise & Fall Time).
7) We use a Bessel beam for a constant resolution in the scanning and flow directions over the full FOV.

Supplementary Note 5: Image Processing Time.

Image formation requires deconvolution of the bead PSF from the raw images, due to aberrations introduced by the skewed imaging geometry with respect to the microfluidic device. The deconvolution takes ~5 s per animal. However, computation was performed on a single core of 3.6 GHz quad-core CPU with 16 GB of RAM. Computation time can either be significantly reduced, or eliminated completely through:

1. **Parallel computation.** After identifying the location of each animal in the dataset, the animals can be processed in parallel, with each animal being handled by one core. The computation time for the population can potentially be reduced to the time it takes a single animal currently (~5 s), given a large number of cores, and fast assignment of tasks to the cores.
2. **FPGA computation.** With knowledge of the system PSF, an FPGA can be designed to quickly process the animals in parallel, potentially reducing the computation time for an entire population to even lower than 5 s[16].
3. **Optical correction or removal of aberrations.** The source of aberrations is the tilted device and coverslip with respect to the excitation beam and imaging plane. Coma and astigmatism can be corrected using a tilted lens or wedge in a conjugate imaging plane[17]. Alternatively, a system can be designed such that the space between the objectives and device is filled with index-matching liquid (using the appropriate objectives and optical design to also counter any additional spherical aberration from the immersion liquid)[18]. These solutions would eliminate aberrations and the need for deconvolution altogether.

Supplementary References

1. Bouchard, M. B. *et al.* Swept confocally-aligned planar excitation (SCAPE) microscopy for high-speed volumetric imaging of behaving organisms. *Nat. Photonics* **9,** 113–119 (2015).

2. Mikami, H. *et al.* Ultrafast confocal fluorescence microscopy beyond the fluorescence lifetime limit. *Optica* **5,** 117 (2018).

3. Nadella, K. M. N. S. *et al.* Random-access scanning microscopy for 3D imaging in awake behaving animals. *Nat. Methods* **13,** 1001–1004 (2016).

4. Fernandez-Alfonso, T. *et al.* Monitoring synaptic and neuronal activity in 3D with synthetic and genetic indicators using a compact acousto-optic lens two-photon microscope. *J. Neurosci. Methods* **222,** 69–81 (2014).

5. Chen, X., Leischner, U., Rochefort, N. L., Nelken, I. & Konnerth, A. Functional mapping of single spines in cortical neurons in vivo. *Nature* **475,** 501–505 (2011).

6. Grewe, B. F., Langer, D., Kasper, H., Kampa, B. M. & Helmchen, F. High-speed in vivo calcium imaging reveals neuronal network activity with near-millisecond precision. *Nat. Methods* **7,** 399–405 (2010).

7. Roorda, R. D., Hohl, T. M., Toledo-Crow, R. & Miesenböck, G. Video-rate nonlinear microscopy of neuronal membrane dynamics with genetically encoded probes. *J. Neurophysiol.* **92,** 609–21 (2004).

8. Cheng, A., Gonçalves, J. T., Golshani, P., Arisaka, K. & Portera-Cailliau, C. Simultaneous two-photon calcium imaging at different depths with spatiotemporal multiplexing. *Nat. Methods* **8,** 139–42 (2011).

9. Duocastella, M. *et al.* Fast Inertia-Free Volumetric Light-Sheet Microscope. *ACS Photonics* **4,** 1797–1804 (2017).

10. Chen, B.-C. *et al.* Lattice light-sheet microscopy: Imaging molecules to embryos at high spatiotemporal resolution. *Science (80-.).* **346,** 1257998–1257998 (2014).

11. Coffman, V. C. & Wu, J.-Q. Counting protein molecules using quantitative fluorescence microscopy. *Trends Biochem. Sci.* **37,** 499–506 (2012).

12. Teich, M. C., Matsuo, K. & Saleh, B. E. A. Excess Noise Factors for Conventional and Superlattice Avalanche Photodiodes and Photomultiplier Tubes. *IEEE J. Quantum Electron.* **22,** 1184–1193 (1986).

13. Sjöback, R. *et al.* Absorption and fluorescence properties of fluorescein. *Acta Part A Mol. Biomol.* **51,** 1–15 (1995).

14. Romer, G. R. B. E. & Bechtold, P. Electro-optic and acousto-optic laser beam scanners. *Phys. Procedia* **56,** 29–39 (2014).

15. Ghorashian, N. Automated Microfluidic Platforms To Facilitate Nerve Degeneration Studies With C . Elegans. (The University of Texas at Austin, 2013).

16. Bromberger, M., Heuveline, V., Rohr, K. & Karl, W. FPGA-accelerated Richardson-Lucy deconvolution for 3D image data. *2016 IEEE 13th Int. Symp. Biomed. Imaging* 132–135 (2016).

17. Mcgorty, R., Xie, D. & Huang, B. High-NA open-top selective-plane illumination microscopy for biological imaging. *Opt. Express* **25,** 17798 (2017).

18. McGorty, R. *et al.* Open-top selective plane illumination microscope for conventionally mounted specimens. *Opt. Express* **23,** 16142–53 (2015).
