# Supplementary material for: Line excitation array detection fluorescence microscopy at 0.8 million frames per second
Source: Nat Commun. 2018 Oct 29;9:4499. doi: 10.1038/s41467-018-06775-0 (PMC6206139; doi:10.1038/s41467-018-06775-0)
Supplement: Supplementary file 3 — Description of Additional Supplementary Files [file 41467_2018_6775_MOESM3_ESM.pdf]

Description of Additional Supplementary Files

File Name: Supplementary Movie 1
Description: Video demonstrating volumetric image capture of the *C. elegans* animal in Figure 1d. Images in the x-y plane (outlined in white) are captured every 1.25 μs by 14 elements of the PMT array. The 2D images in the x-z plane captured by PMT elements #4, 6, and 8 (outlined in red, green, and blue, respectively) are shown to demonstrate the axial sectioning capability of the system. The full volume consists of 631 x-y frames captured in 0.79 ms. Video speed is 16,000× slower than real-time. Scale bar = 50 μm.
